# Establishment of Biobank and Patient-Derived Xenograft of Soft Tissue and Bone Tumors

**DOI:** 10.7759/cureus.74781

**Published:** 2024-11-29

**Authors:** Seiji Okada, Piyanard Boonnate, Jutatip Panaampon, Krittamate Saisuwan, Hiromi Ogata-Aoki, Makoto Abe, Kaoru Hirabayashi, Rumi Nakagawa, Kazutaka Kikuta

**Affiliations:** 1 Division of Hematopoiesis, Joint Research Center for Human Retrovirus Infection, Kumamoto University, Kumamoto, JPN; 2 Laboratory of Environmental Toxicology, Chulabhorn Research Institute, Bangkok, THA; 3 Division of Hematologic Neoplasia, Department of Medical Oncology, Dana-Farber Cancer Institute, Boston, USA; 4 Division of Diagnostic Pathology, Tochigi Cancer Center, Utsunomiya, JPN; 5 Division of Musculoskeletal Oncology and Orthopaedics Surgery, Tochigi Cancer Center, Utsunomiya, JPN

**Keywords:** biobank, bone tumors, patient-derived xenograft models, precision cancer medicine, soft tissue tumours

## Abstract

Soft tissue and bone tumors are rare, and their low frequency and diverse histological types make conducting large-scale clinical trials challenging. Patient-derived xenografts (PDX), entailing implantation of cancer specimens in immunocompromised mice, are emerging as a valuable translational model because PDX keeps the original tumors' character and drug sensitivity. We sequentially transplanted 166 surgical and biopsy specimens from orthopedic surgeries, including 138 soft tissue and bone tumors (81 malignant, 23 intermediate, and 34 benign), 16 metastatic bone tumors, 9 hematological malignancies, and 3 non-tumor tissues. Every specimen was cutaneously transplanted into both flanks of BALB/c Rag-2/Jak3 double deficient (BRJ) mice, and tumor formation was observed for up to 6 months. We defined PDX models as successfully generated if the tumors were passaged more than three times while retaining the histological characteristics of the original tumor. The rates of PDX generation were 28.1% (39/138) for all soft tissue and bone tumors, 42.6% (35/81) for malignant tumors, 4.3% (1/23) for intermediate tumors, and 8.8% (3/34) for benign tumors. Our models of PDX would be a useful platform for soft tissue and bone tumor precision medicine.

## Introduction

Malignant soft tissue and bone tumors are rare, with an incidence of less than six cases per 100,000 people per year, and they consist of over 140 subtypes, categorized into two broad groups: soft-tissue and bone tumors [1-3]. In addition, these tumors can arise in diverse ages with various behavior and molecular etiology. The primary treatment is surgical excision for local lesions; however, the systemic treatment regimens for managing advanced and relapsed diseases have not been established due to their rarity of incidence and diversity of etiology [4].

More than 90% of anti-cancer drug candidates with preclinical activity fail in clinical trials largely due to insufficient efficacy of preclinical animal models [5,6]. Recently, the patient-derived xenograft (PDX) model, established by transplantation of patients’ primary tumors, has been widely used to evaluate drugs and personalized medicine studies [7-9]. Several large-scale biobanks of PDX have been established worldwide, such as EuroPDX in Europe [10], PDX finder at the National Cancer Institute (NCI) [11], the Public Repository of Xenografts (ProXe) at the Dana-Faber Center Institute [12]. In contrast, establishing a nationwide PDX biobank system has been slower in Asian countries, though the PDX biobank at the National Cancer Center Japan was recently established [13].

The primary objective of this study is to establish a biobank and PDX model for soft tissue and bone tumors, which will serve as a platform for precision medicine and drug testing and to share our protocol and the expected results. We report on the trial of a Japanese PDX biobank that provides practical resources to accelerate research into soft tissue and bone tumors by a single institute. It could also clarify evaluating the engraftment rates of different tumor types and assessing the feasibility of long-term tumor passage. Establishing a nationwide and worldwide rare tumor biobank enables us to develop common protocols for the treatment of these rare tumors.

## Materials and methods

Patient selection and consent

A total of 166 operation and biopsy specimens were enrolled and sent from Tochigi Cancer Center to Kumamoto University and transplanted into BRJ mice between 1 December 2020 and 31 December 2022 (25 months). The protocol was approved by the institutional review board (30-78-20-20201119, November 19, 2020), and all patients gave written informed consent. All of the operated and biopsy samples performed at the Division of Musculoskeletal Oncology and Orthopaedics Surgery, Tochigi Cancer Center, with informed consent, were registered in this study. The specimens from Hepatitis B, Hepatitis C, and HIV-1 antigen/antibody-positive patients were excluded from the PDX study to prevent researchers' exposure to infection. The study was conducted with the guidelines following the Helsinki Declaration. The study protocol was approved by the Kumamoto University Animal Care and Use Committee (A2021-052, 1 April 2021) and complied with all applicable regulations, guidance, and local policies of Japan.

Sample collection

Surgically resected tissues or biopsy tissues were obtained from the Division of Musculoskeletal Oncology and Orthopaedics Surgery, Tochigi Cancer Center. The samples were immediately soaked in sample preservation media: DMEM/F12 (048-29785, Fujifilm Wako Pure Chemical, Osaka, Japan) containing 10% fetal bovine serum (FBS, Thermo Fisher Scientific, Waltham, USA) supplemented with 100 µg/mL streptomycin (Fujifilm Wako Pure Chemical), 100 U/mL penicillin G (Meiji Seika Pharma, Tokyo, Japan), and 2.5 µg/mL amphotericin B (Fujifilm Wako Pure Chemical) at 4°C after collection. The samples were anonymized and sent to Kumamoto University by Yamato Transport delivery service at 4°C. Samples are also registered and kept in the Tochigi Cancer Center Cancer Biobank with patients' data.

Sample processing and tumor implantation in immunodeficient mice

After receiving the samples, they were cut into approximately 5-mm^3^ pieces and transplanted into both sides of flanks of 6 to 8-week-old BALB/c Rag-2/Jak3 double deficient (BRJ) mice subcutaneously [8,14]. We used 2-5 mice for transplantation according to the size of the samples (biopsy specimens are relatively small, whereas operation specimens are relatively large). Mice were maintained in specific pathogen-free (SPF) conditions at the Center for Animal Resources Development (CARD), Kumamoto University. All invasive treatments were performed under an anesthesia apparatus (Muromachi Kikai, Tokyo, Japan). Inhalation of isoflurane (Fujifilm Wako Pure Chemical) and acetaminophen (Acelio, Terumo, Tokyo, Japan), including water (1mg/ml), was given for 48 hours post-operation to reduce the pain of the experimental mice.

Monitoring and passage

Mice were monitored weekly for overall health and tumor growth. Tumors were passaged once their size exceeded 1 cm or if a humane endpoint was reached [15,16]. Mice were sacrificed by cervical dislocation under anesthesia, and the tumors were taken away from mice. Removed tumors were cut into approximately 5-mm^3^ and immediately transplanted into BRJ mice. The rest of the tumors were stored as samples for re-transplantation in freezing media (Bambanker, NIPPON Genetics, Tokyo, Japan) in a liquid nitrogen tank for the preparation of samples for pathological examination, and for preparing DNA and RNA. The first tumor generated was more than 1 cm in diameter and was designated as Passage 1 (P1). It was removed from the mice and was serially transplanted for further generations (P2, P3, etc.) up to the fifth generation (P5) [17].

Immunohistochemistry

Tumor tissues were stained with Hematoxylin-Eosin (H&E), human CD45 (#13917, clone D9M8I, Cell Signaling Technology), and human COX IV (#4850, clone 3E11, Cell Signaling Technology) to verify that the tumors were not replaced by lymphoma and murine tumors. [13,18]. To confirm the diagnosis of soft tissue and bone tumor, AE1/3 (Clone: AE1, AE3, Nichirei, Tokyo, Japan), desmin (D33, Nichirei, Tokyo, Japan), S-100 (100, Dako), CD34 (NU-4A1, Nichirei, Tokyo, Japan), and vimentin (V9, Nichirei, Tokyo, Japan) were used.

## Results

Tochigi cancer center and biobank

Tochigi Cancer Center established Tochigi Cancer Biobank in April 2021, which collects, stocks, and provides patient’s tumor samples, including living samples frozen in cryopreservation media (Cell Banker, Takara, Kyoto, Japan), clinical specimens (blood, urine, non-tumor part of tumor tissues), pathology, image data and patients’ clinical data (https://cancerbiobank.jp/en/). Tochigi Cancer Biobank focuses on rare cancers, especially soft tissue and bone tumors, because it is especially important to establish the tumor bank. The content of the tumor bank can provide essential material for current and future research, such as developing novel therapy, analyzing the character of the tumor, and the prognostic or predictive tumor markers. Most of the northern Kanto region's soft tissue and bone tumor patients (population: 7 million) are introduced to Tochigi Cancer Center. Tochigi Cancer Center gathered 267 soft tissue and bone tumor samples in two years (April 2021-March 2023).

Enrollment progress

**Figure 1:**
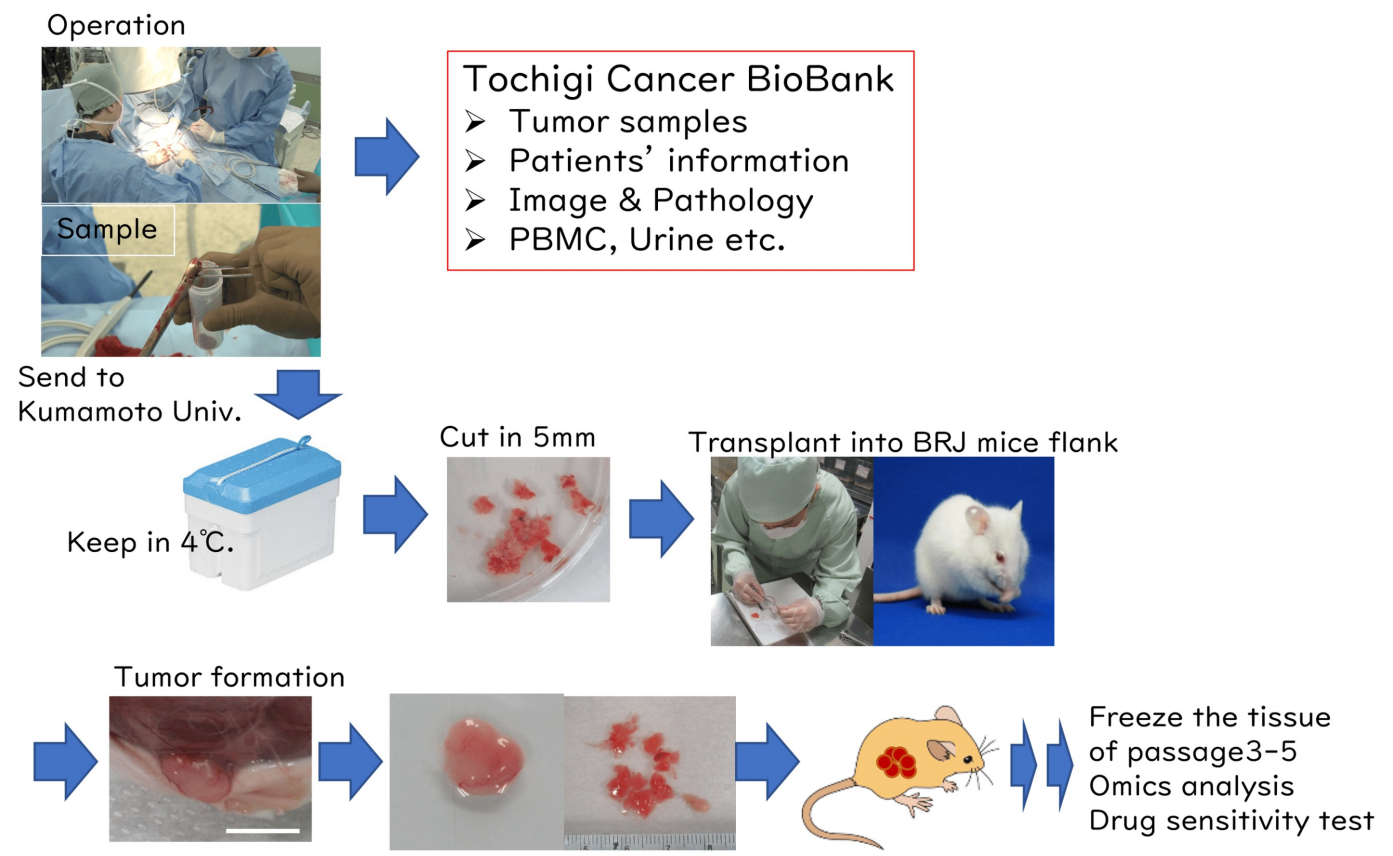
Establishment of patient-derived xenograft (PDX) by the samples from Tochigi Cancer Center. (a) Samples from patients are gathered after obtaining written informed consent. (b) Specimens are immediately soaked into sample preservation media and transported to Kumamoto University by transport delivery service at 4°C. (c) The specimens were cut into approximately 5-mm^3^ pieces and transplanted into both 6 to 8-week-old BRJ mice flanks. (d) Mice were observed weekly for health conditions and tumor growth, and tumor passage was conducted when the tumor size reached more than 1 cm in diameter. Removed tumors were cut into 5-mm3 and passaged for the next generation, and the rest of the tumors were stored for re-transplantation in freezing media (Bambanker, NIPPON Genetics, Tokyo, Japan) at a liquid nitrogen tank and for the preparation of samples for pathological blocks and fresh frozen samples.

A total of 166 operation and biopsy specimens were enrolled and sent to Kumamoto University from Tochigi Cancer Bank, transplanted into BRJ mice between 1 December 2020 and 31 December 2022 (25 months). The samples were immediately soaked into preservation media and sent by Yamato Delivery Service Co. It took two nights to arrive at Kumamoto University from Tochigi Cancer Center since the distance between Tochigi and Kumamoto is approximately 1,000 km. We performed preliminary experiments to send the specimen and analyzed survival by culture of the specimen before starting the project. The samples were cut into approximately 5-mm^3^ pieces and transplanted into BRJ mice (Figure 1). Of the 166 specimens enrolled, 141 specimens were soft tissue or bone tumors, 15 specimens were metastatic bone tumors (lung cancer 3, malignant melanoma 3, renal cell carcinoma 3, tumor of unknown origin 1, breast cancer 1, prostate cancer 1), and 6 cases were hematological malignancies (lymphoma, multiple myeloma, and plasmacytoma). Since biopsies were performed for diagnosis of the mass, the pathological diagnosis was not confirmed at the time of transplantation, and three cases were diagnosed with no tumor. Of the 138 soft tissue and bone tumors, 81 cases were malignant, 23 cases were intermediate, and 34 cases were benign tumors.

Establishment of PDX and engraftment rate

The status of the establishment of PDX is shown in Table 1, where the percentage of transplantable tumors of the total assessable specimens was calculated as the engraftment rate. The engraftment rate for soft tissue and bone tumors was 28.1% (39/138), 42.6% (35/81) for malignant tumors, 4.3% (1/23) for intermediate tumors, and 8.8% (3/34) for benign tumors. One established case of intermediated malignancy was dermatofibrosarcoma protuberans (DFSP).

**Table 1:** Number of transplanted samples and establishment ratio of PDX. *Biological potential (grading) is based on the 2020 WHO classification of tumors of soft tissue/bone [1]. PDX: patient-derived xenograft.

	Biological potential (grading) *	Number of cases transplanted (%)	Number of instances established PDX	Establishment ratio of PDX (%)
Soft tissue and bone tumor	Malignant	81 (48.8)	35	42.6
	Intermediate	23 (13.9)	1	4.3
	Benign	34 (20.5)	3	8.8
	Total	138 (83.1)	39	28.1
Metastatic tumor		16 (9.6)	7	43.4
Hematological malignancy		9 (5.4)	1	11.1
No tumor		3 (1.8)	0	0
Total		166	47	28.3

The three cases of benign tumors were hemangioma, chondroma, and nodular/proliferative fasciitis. The engraftment rate of metastatic bone tumors was 43.8% (7/16) overall; 100% in malignant melanoma (3/3), lung cancer (2/2), and tumor of unknown origin (1/1); and 16.7% in renal cell carcinoma (1/6), 0% in breast cancer (0/2), thyroid cancer (0/1), and prostate cancer (0/1) (Table 2). 

**Table 2:** Establishment of PDX from metastatic bone tumors. PDX: patient-derived xenograft.

	Diagnosis	Number of cases obtained	Percentage of cases of metastatic bone tumors	Number of PDX established / Number of cases (%)
1	Renal cell carcinoma	6	37.5	1/6 (16.7%)
2	Melanoma	3	18.75	3/3 (100%)
3	Lung cancer	2	12.5	2/2 (100%)
4	Breast cancer	2	12.5	0/2 (0%)
5	Carcinoma of unknown origin	1	6.25	1/1 (100%)
6	Thyroid cancer	1	6.25	0/1 (0%)
7	Prostate cancer	1	6.25	0/1 (0%)
	Total	16	100	7/16 (43.8%)

Soft tissue and bone tumors are rare cancers with a wide range of histological types. Table 3 shows the list of a higher number of cases obtained in our study. The number of samples has a little bias by small number of samples, but are nearly related to the incidence of soft tissue and bone tumors in Japan [2,3]. Myxofibrosarcoma, myxoid liposarcoma, and undifferentiated pleomorphic sarcoma had a relatively high number of cases and a higher rate of establishing PDX (Table 3). Dedifferentiated liposarcoma, Ewing sarcoma, and osteosarcoma also have a high rate of establishing PDX. On the other hand, chondrosarcoma and giant cell bone tumors (intermediate) and schwannoma (benign) could not make PDX. Pathologists confirmed the retention of morphology and phenotype of the established PDX with hematoxylin-eosin staining and immunohistochemistry.

**Table 3:** Establishment of PDX from soft tissue and bone tumors. PDX: patient-derived xanograft, listed from the higher number of samples obtained and rate of established PDX.

	Diagnosis	Biological potential	Number of cases	PDX
1	Myxofibrosarcoma (MFS)	malignant	１2	6 （50.0%）
1	Myxoid liposarcoma	malignant	12	4 （33.3％）
3	Undifferentiated pleomorphic sarcoma (UPS)	malignant	10	5 （50.0％）
4	Chondrosarcoma	intermediate	10	0 （0％）
4	Giant cell tumor of bone (GCTB)	intermediate	9	0 （0％）
6	Dedifferentiated liposarcoma (DDLP)	malignant	7	4 （57.1％）
7	Ewing sarcoma	malignant	6	2 （33.3%)
8	Osteosarcoma	malignant	5	3 （60.0%)
8	Schwannoma	benign	5	0 （0％）
10	Dermatofibrosarcoma protuberans (DFSP)	intermediate	４	1 （25.0%）

Assessment of the time of the growth of PDX tumor

When the tumors developed more than 1 cm in diameter, mice were sacrificed, and tumors were transplanted into the next mice. One case of undifferentiated pleomorphic sarcoma (UPS) was confirmed as Epstein-Barr virus-infected B cell lymphoma by morphology and anti-CD20 Ab staining. One case of UPS was confirmed as a mouse-derived tumor by mitochondria staining. One case of a malignant peripheral nerve sheath tumor (MPNST) ceased growth at the P1 stage. Overall, 38 cases of bone and soft tissue tumors successfully formed subcutaneous tumors, which were subsequently transplanted into more than three generations. The tumors retained the characteristics of the original tumor, as confirmed through hematoxylin and eosin staining and histochemical analysis using markers such as AE1/3, desmin, S-100, CD34, and vimentin.

**Figure 2:**
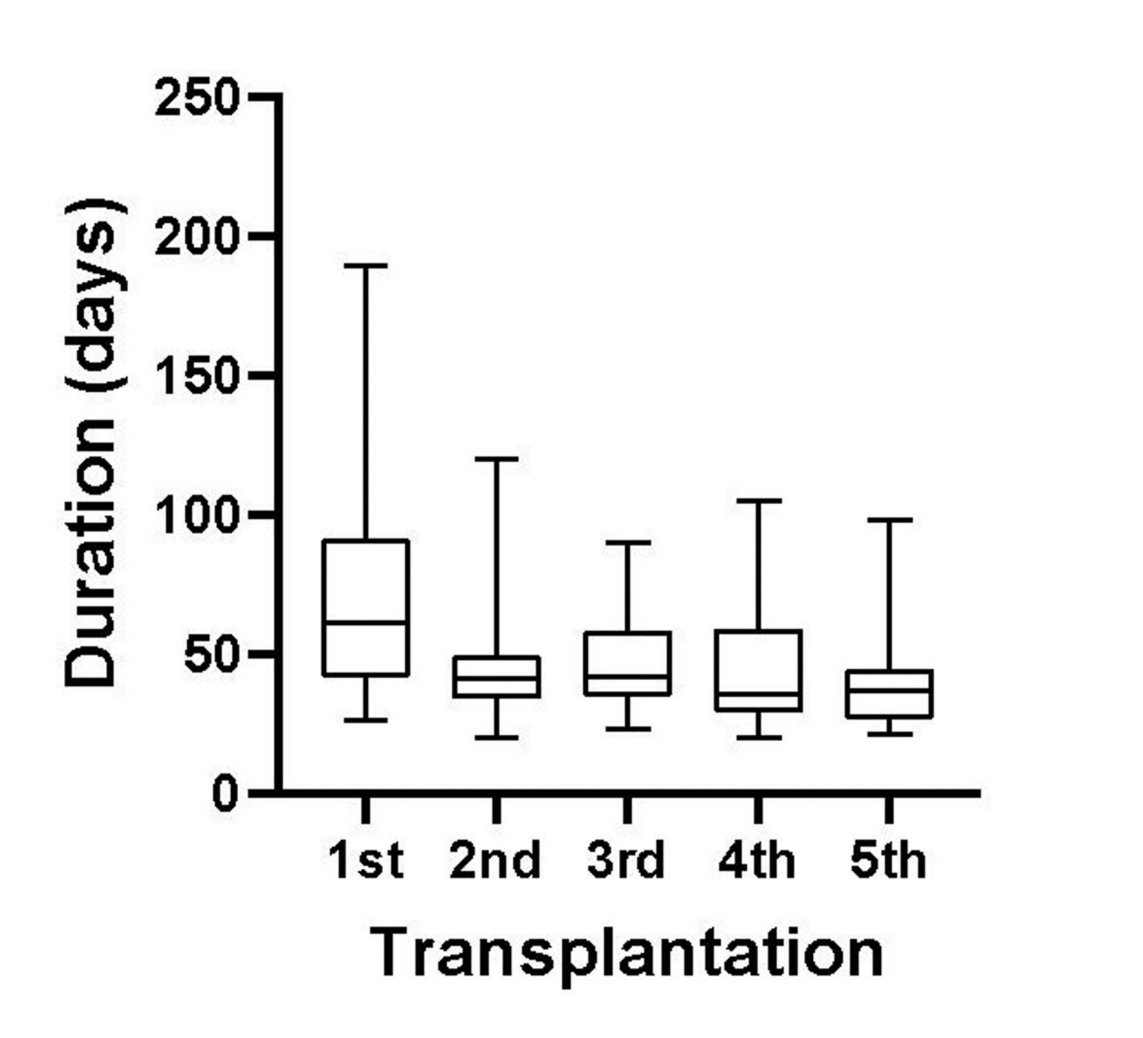
Comparison of PDX growth duration during serial transplantation. PDX: patient-derived xanograft.

The duration of the 1st passage (P1) tumor formations ranged from 26 to 189 days (median 61 days). The median duration of 2nd to 5th passages (P2-P5) was 35.5-42 days, ranging from 20 to 105 days, and the mean duration of PDX growth in P1, P2, P3, P4, and P5 was 76.9, 46.4, 47.1, 44.2 and 40.6 days, respectively. Thus, although the duration of P1 varied and took more term, P2-P5 PDXs were established on an average of 40-50 days. The total duration to establish until practically useful PDX (until P3) was 170.1±576.9 days (Median 147 days, maximum 377 days, minimum 83 days) (Figure 2), which is consistent with other PDXs [13,17].

## Discussion

This is the first report, to the best of our knowledge, of systemic and consecutive PDX establishment from orthopedics surgery or biopsy samples by a single institute in Japan. The overall establishment rate of soft tissue and bone tumors was 27.5%, with a rate of 42.2% for malignant soft tissue and bone tumors, 4.3% for intermediate soft tissue and bone tumors, and 8.8% for benign tumors. Although it needed more than 48 hours from sample harvest to transplantation, the rate of PDX establishment was consistent or rather better than the other reports. In addition, since Tochigi Biobank keeps the patients’ information, including diagnostic images, pathology, and samples (peripheral blood, urine, tumor samples, etc.), our PDX biobank has the advantage for evaluating multiple valuation approaches.

Athymic nude mice have been most frequently used as the host for establishing primary PDX models of soft tissue and bone tumors [19], which is characterized by the lack of mature and functional T lymphocytes and T cell-dependent immune response: in addition, the phenotype of lacking hair on the skin has the advantage of observing the growth of transplanted human tumors [8]. However, nude mice have intact NK cells, which kill tumor cells and leakage of T cells. Recently, NOD/Scid-based mice have been widely used in the transplantation of human tumors, as NOD/Scid mice have defective innate immunity in addition to the lack of T and B lymphocytes [20]. More recently, NOD/Scid commonγ^null ^(NOG and NSG) and NOD/Scid Jak3^null ^(NOJ) mice, which refer to the most immune-deficient mouse strains, are frequently used for generating PDX models. However, the Prkdc*^scid^* mutation results in the dysfunction of the PRKDC gene, which encodes a protein responsible for DNA repair in all tissues, indicating that Scid mutation background mice are not appropriate to test reagents that inhibit DNA damage repair (DDR) or DNA-damaging drugs. We established BALB/c Rag-2/Jak3 double deficient (BRJ) mice to overcome the disadvantages of these immunodeficient mice [14]. BRJ mice were shown as the appropriate host of human hematopoietic stem cells, peripheral blood mononuclear cells, and human tumor cell lines. It was also shown that BRJ mice showed as high as 75% (established 12 cases out of 16 cases transplanted) efficiency in establishing cholangiocarcinoma PDX [17]. In this study, the establishment ratio of PDX was 27.5% in total and 42% in malignant soft tissue and bone tumors, which is a relatively higher rate compared with the data from the National Cancer Center of Japan(J-PDX) [13]. The tumor samples were immediately transplanted into NSG mice at J-PDX, whereas our samples were transplanted two days after harvested (Figure 1). Thus, we showed here that soft tissue and bone tumor PDX can be established from the samples even after 2 days, indicating that soft tissue and bone tumor samples can be gathered from nationwide hospitals and it is possible to establish large-scale soft tissue and bone tumor PDX Biobank in Japan, which contribute the progress of soft tissue and bone tumor research.

Recently, the NSG and NOG strain is considered an essential donor for developing PDX mice [21], which show high immunodeficiency at the same level [20, 22, 23]. However, a significant number of tumor-transplanted mice are known to develop lymphoproliferative disease by Epstein-Barr virus (EBV) infected B lymphocytes, especially transplantation of gastrointestinal tumors (gastric cancer and colon cancer) and metastatic tumors for lymph nodes [18,24]. Yagishita et al. showed a significant number of rare cancers/sarcoma also developed lymphoma (16 cases out of 301 cases: 5.3%) [13]. In our study, only one case among 166 transplanted cases (0.006%) developed EB virus-mediated lymphoma. We assume that this is according to the genetic background of mice, as NOD/Scid mice are known to develop spontaneous lymphoma [25,26]. Although NOG and NSG mice have no spontaneous lymphoma, the microenvironment of these mice may fit to develop lymphoma. Corso et al. showed rituximab treatment prevents the onset of lymphoma in gastric cancer PDX [27].

Major PDX banks focus mainly on major cancers such as GI tract, breast, and lung cancer because they have more benefits for patients [10-12]. However, we also need to establish standard treatment for rare but important tumors [9,28]. Especially since soft tissue and bone tumors are developing in young to older people, and standard therapy except surgery is not established yet, patient-derived models play important roles. Many research groups are now trying to establish PDX models [28-30]. However, opened PDX banks of bone and soft tissue tumors currently do not cover all types [11]. We expect our Biobank and PDX will contribute to a better understanding of these tumors’ pathophysiology and to the development of novel therapeutics.

In this study, we established a single-institute Biobank of soft tissue and bone tumors. In general, multi-institute clinical studies have more advantages when recruiting the samples. However, since the number of patients is limited and specialists in soft tissue and bone tumors are limited in Japan, a single-institute Biobank can manage rare tumors because the patients are gathered in the hospital specializing in soft tissue and bone tumors for treatment. It is relatively easy to gather the patients in specific hospitals such as Tochigi Cancer Center. Furthermore, since the staff (orthopedic surgeons, pathologists, medical technicians, Biobank staff, etc.) belongs to the same institute, the staff can share the property of the importance of Biobank, and it is easier to exchange information face-to-face and follow up with the patients. In our study, we had a relatively high engraftment rate, which contributed by the selection of the tumor site by the skillful surgeons as well as keeping the appropriate conditions until transplantation. Thus, single-institute BioBank has several advantages for registration and management and is practically very useful with PDX information in the case of rare tumors. On the other hand, since the pathological subtypes of soft tissue and bone tumors are diverse (more than 100 subtypes in soft tissue tumors and 50 subtypes in bone tumors) [1], it has the limitation to gather all of the phenotypes of these tumors in a single institute. In this aspect, we need to establish a nationwide and worldwide Biobank in the near future. Biobank with PDX would be a useful platform for the translational research of soft tissue and bone tumors.

## Conclusions

Herein, we reported our new soft tissue and bone tumor Biobank at a single institute level with patient-derived xenograft (PDX). As patient-derived xenografts (PDXs) maintain the diversity, heterogeneity, and characteristics of patients’ tumors, they are now anticipated to serve as an efficient bridge between pre-clinical and clinical studies. We could establish a relatively high ratio of PDX from malignant soft tissue and bone tumors (39 cases/138 cases: 28.1%). This biobank has value as a preclinical tool for further drug development. Since major pharmaceutical companies do not focus on rare tumors according to cost-performance and clinical studies are difficult due to the limited number of patients, PDX and Biobank would be useful platforms for the translational research of soft tissue and bone tumors especially the evaluation of novel treatment of these rare tumors.

## References

[1] WHO Classification of Tumours Editorial Board (2020). Soft Tissue and Bone Tumours. WHO classificstion of tumours. https://publications.iarc.fr/Book-And-Report-Series/Who-Classification-Of-Tumours/Soft-Tissue-And-Bone-Tumours-2020.

[2] Ogura K, Higashi T, Kawai A (2017). Statistics of bone sarcoma in Japan: report from the bone and soft tissue tumor registry in Japan. J Orthop Sci.

[3] Ogura K, Higashi T, Kawai A (2017). Statistics of soft-tissue sarcoma in Japan: report from the bone and soft tissue tumor registry in Japan. J Orthop Sci.

[4] Xu H, Zheng H, Zhang Q (2023). A multicentre clinical study of sarcoma personalised treatment using patient-derived tumour xenografts. Clin Oncol (R Coll Radiol).

[5] Alteri E, Guizzaro L (2018). Be open about drug failures to speed up research. Nature.

[6] DiMasi JA, Feldman L, Seckler A, Wilson A (2010). Trends in risks associated with new drug development: success rates for investigational drugs. Clin Pharmacol Ther.

[7] Aparicio S, Hidalgo M, Kung AL (2015). Examining the utility of patient-derived xenograft mouse models. Nat Rev Cancer.

[8] Okada S, Vaeteewoottacharn K, Kariya R (2019). Application of highly immunocompromised mice for the establishment of patient-derived xenograft (PDX) models. Cells.

[9] Abdolahi S, Ghazvinian Z, Muhammadnejad S, Saleh M, Asadzadeh Aghdaei H, Baghaei K (2022). Patient-derived xenograft (PDX) models, applications and challenges in cancer research. J Transl Med.

[10] Hoang NT, Acevedo LA, Mann MJ, Tolani B (2018). A review of soft-tissue sarcomas: translation of biological advances into treatment measures. Cancer Manag Res.

[11] Conte N, Mason JC, Halmagyi C (2019). PDX Finder: A portal for patient-derived tumor xenograft model discovery. Nucleic Acids Res.

[12] Townsend EC, Murakami MA, Christodoulou A (2016). The public repository of xenografts enables discovery and randomized phase ii-like trials in mice. Cancer Cell.

[13] Yagishita S, Kato K, Takahashi M (2021). Characterization of the large-scale Japanese patient-derived xenograft (J-PDX) library. Cancer Sci.

[14] Ono A, Hattori S, Kariya R (2011). Comparative study of human hematopoietic cell engraftment into BALB/c and C57BL/6 strain of rag-2/jak3 double-deficient mice. J Biomed Biotechnol.

[15] Wallace J (2000). Humane endpoints and cancer research. ILAR J.

[16] Stokes WS (2002). Humane endpoints for laboratory animals used in regulatory testing. ILAR J.

[17] Vaeteewoottacharn K, Pairojkul C, Kariya R (2019). Establishment of highly transplantable cholangiocarcinoma cell lines from a patient-derived xenograft mouse model. Cells.

[18] Dieter SM, Giessler KM, Kriegsmann M (2017). Patient-derived xenografts of gastrointestinal cancers are susceptible to rapid and delayed B-lymphoproliferation. Int J Cancer.

[19] Lu W, Chao T, Ruiqi C, Juan S, Zhihong L (2018). Patient-derived xenograft models in musculoskeletal malignancies. J Transl Med.

[20] Shultz LD, Lyons BL, Burzenski LM (2005). Human lymphoid and myeloid cell development in NOD/LtSz-scid IL2R gamma null mice engrafted with mobilized human hemopoietic stem cells. J Immunol.

[21] Okada S, Vaeteewoottacharn K, Kariya R (2018). Establishment of a patient-derived tumor xenograft model and application for precision cancer medicine. Chem Pharm Bull (Tokyo).

[22] Ito M, Hiramatsu H, Kobayashi K (2002). NOD/SCID/gamma(c)(null) mouse: an excellent recipient mouse model for engraftment of human cells. Blood.

[23] Barve A, Casson L, Krem M, Wunderlich M, Mulloy JC, Beverly LJ (2018). Comparative utility of NRG and NRGS mice for the study of normal hematopoiesis, leukemogenesis, and therapeutic response. Exp Hematol.

[24] Bondarenko G, Ugolkov A, Rohan S (2015). Establishment of a patient-derived tumor xenograft model and application for precision cancer medicine. Neoplasia.

[25] Custer RP, Bosma GC, Bosma MJ (1985). Severe combined immunodeficiency (SCID) in the mouse. Pathology, reconstitution, neoplasms. Am J Pathol.

[26] Chiu PP, Ivakine E, Mortin-Toth S, Danska JS (2002). Susceptibility to lymphoid neoplasia in immunodeficient strains of nonobese diabetic mice. Cancer Res.

[27] Corso S, Cargnelutti M, Durando S (2018). Rituximab treatment prevents lymphoma onset in gastric cancer patient-derived xenografts. Neoplasia.

[28] Igarashi K, Kawaguchi K, Murakami T (2020). Patient-derived orthotopic xenograft models of sarcoma. Cancer Lett.

[29] Cornillie J, Wozniak A, Li H (2019). Establishment and characterization of histologically and molecularly stable soft-tissue sarcoma xenograft models for biological studies and preclinical drug testing. Mol Cancer Ther.

[30] Higuchi T, Igarashi K, Yamamoto N (2021). Osteosarcoma patient-derived orthotopic xenograft (pdox) models used to identify novel and effective therapeutics: a review. Anticancer Res.

